# CA 19-9 as a biomarker in advanced pancreatic cancer patients randomised to gemcitabine plus axitinib or gemcitabine alone

**DOI:** 10.1038/sj.bjc.6605243

**Published:** 2009-09-01

**Authors:** H S Wasan, G M Springett, C Chodkiewicz, R Wong, J Maurel, C Barone, B Rosbrook, A D Ricart, S Kim, J-P Spano

**Affiliations:** 1 Department of Cancer Medicine, Hammersmith Hospital, Du Cane Road, London W12 0HS, UK; 2 H. Lee Moffitt Cancer Center and Research Institute, 12902 Magnolia Drive, Tampa, FL 33612, USA; 3 Cancer Care Manitoba, St. Boniface General Hospital, 409 Tache Avenue, Winnipeg, Manitoba R2H 2A6, Canada; 4 Hospital Clinic de Barcelona, CIBEREHD, Villarroel 170, Barcelona 08036, Spain; 5 Catholic University Sacro Cuore, Largo F Vito, Rome 1–00168, Italy; 6 Pfizer Oncology, Science Center Drive, CA 92121, San Diego, USA; 7 Hôpital de la Pitié Salpêtrière, 47–83 Boulevard de l'Hôpital, 75651 Paris Cedex 13, France

**Keywords:** advanced pancreatic cancer, CA 19-9, biomarkers, axitinib

## Abstract

**Background:**

Response assessment in advanced pancreatic cancer (APC) is difficult and predictive markers are needed. There are insufficient data on the value of carbohydrate antigen 19–9 (CA 19-9) and cytostatic-targeted therapies. Axitinib, a selective vascular endothelial growth factor (VEGF) receptors 1, 2, 3 inhibitor, may increase overall survival (OS) in APC.

**Methods:**

We assessed serum CA 19-9, clinical outcomes and diastolic blood pressure (dBP) in APC patients receiving gemcitabine plus axitinib (Gem+A) or gemcitabine alone.

**Results:**

In the total population (*N*=95), median OS was significantly longer in patients with baseline CA 19-9 values at or below the median than in those with values above it (12.2 months [95% confidence interval (CI), 8.6–16.6%] *vs* 5.0 months [95% CI, 3.9–5.7%]; *P*<0.0001). This also reached significance in the Gem+A arm (median OS, 12.5 months [95% CI, 8.6–16.6%] *vs* 4.9 months [95% CI, 3.6–5.6%]; *P*<0.0001). Patients with any dBP>90 mmHg had significantly longer OS than those who did not. However, there was no predictive significance of CA 19-9.

**Conclusion:**

Baseline CA 19-9 levels had prognostic value for OS, but caution is advised in interpreting CA 19-9 as a predictive biomarker for novel cytostatic agents such as VEGF-targeted therapies in phase II studies.

Monitoring objective responses to systemic therapy using conventional imaging can be difficult in patients with advanced pancreatic cancer (APC) whose disease is located predominantly at the primary site (Rothenberg *et al*, 1996). These techniques may underestimate the response to systemic therapy, as the image incorporates tumour desmoplasia, which is prevalent in pancreatic tumour growth and is unlikely to be affected by systemic therapy. In addition, favourable objective response rates in early studies of new targeted agents or combinations have not been predictive of benefit in phase III studies (Xiong *et al*, 2004; Kindler *et al*, 2005, 2007; Phillip *et al*, 2007). Alternative predictive methods for monitoring therapy benefit and predicting prognosis in early phase studies are urgently needed especially as vascular endothelial growth factor (VEGF)-targeted strategies do not seem to contribute significantly to classical objective assessments in terms of complete response+partial response (PR).

As its initial characterisation in 1979 (Koprowski *et al*, 1979, 1981), the role of the tumour-associated carbohydrate antigen 19-9 (CA 19-9) as a serum marker for tumours has been widely studied, particularly in patients with pancreatic cancer. CA 19-9 is the sialylated Lewis (Le)^a^ blood group antigen (Magnani *et al*, 1982), and cannot be synthesised in individuals with a Le^a–b–^ phenotype (5% of the population) (Tempero *et al*, 1987). Serum CA 19-9 seems to show greatest specificity for pancreatic cancer, being elevated in approximately 70–80% of patients (Del Villano *et al*, 1983; Jalanko *et al*, 1984; Haglund *et al*, 1986; Safi *et al*, 1989, 1997). Despite these findings, CA 19-9 is not currently recommended as a routine diagnostic or screening test for pancreatic cancer (Frebourg *et al*, 1988; Locker *et al*, 2006), as its specificity and sensitivity are inadequate for accurate diagnosis. Serum CA 19-9 may also be increased in a number of benign conditions, including benign hepatobiliary diseases, and in biliary obstruction (Haglund *et al*, 1986; Frebourg *et al*, 1988).

The prognostic value of peri-operative CA 19-9 levels and its role as an indicator of asymptomatic recurrence have been investigated in patients with resectable pancreatic cancer (Jiang *et al*, 2004; Ferrone *et al*, 2006), and a recent phase III trial confirmed the prognostic value of post-resection CA 19-9 levels in patients undergoing surgery with curative intent (Berger *et al*, 2008). However, the greatest potential lies in exploiting CA 19-9 as a biomarker in patients with advanced inoperable pancreatic cancer, who comprise at least 80% of those diagnosed (National Cancer Institute, 2008). Several studies have found baseline CA 19-9 levels to be an independent prognostic factor for survival in patients with APC (Saad *et al*, 2002; Micke *et al*, 2003; Louvet *et al*, 2005; Maisey *et al*, 2005; Hess *et al*, 2008; Park *et al*, 2008). An association between reduction in CA 19-9 levels during treatment and prolonged survival has also been reported (Halm *et al*, 2000; Saad *et al*, 2002; Stemmler *et al*, 2003; Ziske *et al*, 2003; Ko *et al*, 2005). However, none of the large randomised studies has reported on the specific impact of targeted cytostatic therapies on CA 19-9 levels as a predictive biomarker.

This retrospective analysis describes the kinetics and the prognostic value of CA 19-9 and investigates the association with clinical outcome and diastolic blood pressure (dBP) in patients with APC receiving gemcitabine plus axitinib (an oral, potent inhibitor of VEGF receptors [VEGFR] 1, 2, 3; Gem+A) or gemcitabine alone (Gem) in a randomised phase II trial (Spano *et al*, 2008). We have already shown that dBP may be a predictive biomarker in those benefiting from axitinib (Spano *et al*, 2008); in the present analysis, we also explore further the correlation between changes in CA 19-9 levels and dBP.

## Materials and methods

### Patients

Eligible patients were aged ⩾18 years with histologically/cytologically confirmed, locally advanced (unresectable) or metastatic pancreatic adenocarcinoma, and had not received any prior systemic therapy for advanced disease. Other inclusion criteria included Eastern Cooperative Oncology Group performance status (ECOG PS) of 0–2 and adequate hepatic, renal and bone marrow function (bilirubin ⩽1.5 × the upper limit of normal). The absence of biliary obstruction at baseline minimised the possibility of false reflection of disease activity. Patients were excluded if they had received prior treatment with gemcitabine or anti-angiogenic agents and were also excluded in the case of pregnancy or lactation, prior cerebrovascular accident, major surgery within 4 weeks of starting treatment, brain metastases, active second malignancy, uncontrolled intercurrent illness, urine protein ⩾500 mg in 24 h or ongoing uncontrolled hypertension. Patients with current or anticipated need for cytochrome P450 CYP1A2 inducers or CYP3A4 inhibitors or inducers were not eligible. The phase II study was approved by the institutional review board at each of the participating centres and was performed in accordance with the Declaration of Helsinki and Good Clinical Practice Guidelines.

### Study design and treatment

The phase II, open-label, multicentre study was designed to compare overall survival (OS) in patients with APC who were randomly assigned in a 2 : 1 ratio to receive Gem+A or Gem. Stratification factors included disease extent (locally advanced *vs* metastatic) and ECOG PS (0/1 *vs* 2). Tumour response was determined using Response Evaluation Criteria in Solid Tumors (RECIST) (Therasse *et al*, 2000). Gemcitabine was administered at a dose of 1000 mg m^–2^ by 30-min intravenous infusion on days 1, 8 and 15 in 4-week cycles in both treatment arms; those randomised to Gem+A also received axitinib 5 mg twice a day, continuously. Toxicity-based dose reductions were pre-specified in the protocol.

### Assessment of CA 19-9

Blood samples for measurement of CA 19-9 were collected at baseline (within 14 days before the start of treatment) and at least every 4 weeks during treatment with either Gem+A or Gem. CA 19-9 levels were determined at individual trial centres using each centre's standard assay and methodology. Most frequently reported reference range was 0–37 U ml^–1^. CA 19-9 measurements were routinely performed at the same laboratory, ensuring reasonable intra-patient consistency.

### Assessment of blood pressure

Blood pressure was measured as part of a physical examination (which also included cardiac function and laboratory analyses of haematology and urinalysis) at baseline, with repeated assessments made on day 1 of each gemcitabine cycle in both treatment arms. A follow-up assessment was carried out 28 days after the last treatment dose.

### Statistical considerations

Overall study sample size was determined by the primary end point of OS. Descriptive statistics were used to record baseline CA 19-9 levels and the percentage change of this biomarker relative to baseline. In successive analyses, patients in each treatment arm were grouped according to the maximum level of CA 19-9 reduction (or response) during treatment (⩾25% decline *vs* <25% decline; ⩾50% decline *vs* <50% decline). Sustained biomarker decline (i.e. confirmation by two measurements spaced at least 28 days apart) was not required. Different to the analysis by Ko *et al* (2005), patients with only one CA 19-9 measurement were excluded from the CA 19-9 reduction analysis as non-responders. These were not categorised as non-decliners.

Median baseline CA 19-9 level was ascertained for each treatment arm. The log-rank test was used to compare OS in patients with baseline levels that fell above or below the median level (based on the analysis by Maisey *et al*, 2005), and to compare OS and progression-free survival (PFS) in patient groups below and above each of the above CA 19-9 response thresholds, by treatment arm. The Kaplan–Meier method was used to generate survival curves according to baseline CA 19-9 levels and to CA 19-9 response during treatment. A Cox proportional hazards model using a ⩾25% reduction in CA 19-9 level as a time-dependent covariate was used to examine the relationship between survival and reduced CA 19-9 levels with time.

Patients were also grouped according to maximum dBP achieved on study (<90 mmHg *vs* ⩾90 mmHg). Within the subgroups, the median drop in CA 19-9 from baseline to nadir was measured and compared using a Wilcoxon rank sum test. The Kaplan–Meier method was used to estimate median OS for each subgroup.

## Results

Of the 103 patients enrolled in the study, 95 patients (65 and 30 patients randomised to Gem+A and to Gem, respectively) had a baseline CA 19-9 sample higher than the reference range (based on the lower limit) and were considered eligible for analysis. Patient baseline characteristics were well matched between the treatment arms (Table 1).

### CA 19-9 kinetics

Median baseline CA 19-9 values were 1096.0 U ml^–1^ (range, 1.6–352 179.6) and 1709.5 U ml^–1^ (range, 16.0–122 163.0) in the Gem+A and Gem arms, respectively. Fifty-three patients randomised to the Gem+A arm had ⩾1 CA 19-9 measurement during treatment and achieved a median nadir value of 136.0 U ml^–1^ (range, 1.0–40 175.0); the median time to nadir was 70 days (range, 15–251). Among the 22 patients with CA 19-9 measurements during treatment with Gem, the median nadir was 471.5 U ml^–1^ (range, 0.8–53 115.3), with a median time to nadir of 79.5 days (range, 26–287). In the Gem+A and Gem arms, 37 patients (86%) and 13 patients (65%), respectively, achieved a ⩾25% reduction in the level of CA 19-9 during treatment when compared with baseline; these proportions were not significantly different (*P*=0.092). Among patients on the Gem+A arm, CA 19-9 levels during treatment fell by ⩾50% compared with baseline in 31 patients (72%) and by ⩾75% in 15 patients (35%); 12 patients (60%) and 6 patients (30%) on the Gem arm achieved on-treatment CA 19-9 reductions of ⩾50% and ⩾75%, respectively. Differences were not statistically significant between treatment arms. Twenty patients had CA 19-9 measurements only at baseline and, per definition, were classified as non-responders and excluded from the CA 19-9 analysis.

### Relationship between CA 19-9 and clinical efficacy

**Figure 1:**
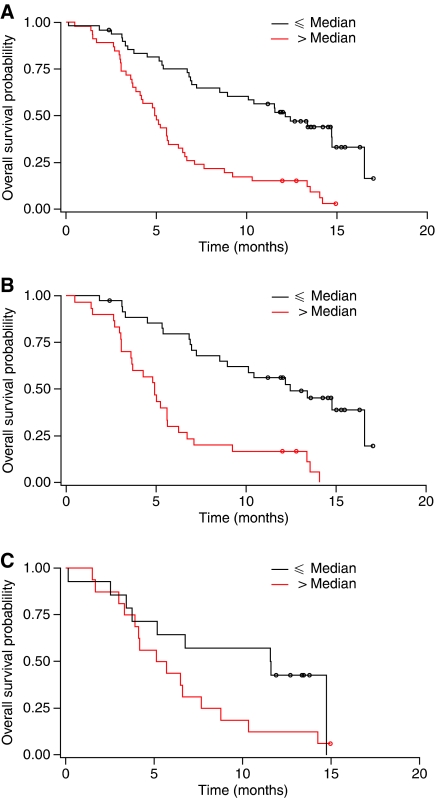
CA 19-9 as a prognostic factor: Kaplan–Meier curves for OS according to baseline CA 19-9 value (⩽ and > the median) (**A**) in both treatment groups combined, (**B**) for the Gem+A arm only and (**C**) for the Gem arm only.

In the overall population (i.e. both treatment groups combined), median OS was significantly longer in patients with a baseline CA 19-9 level equal to or below the median value than in patients with baseline CA 19-9 above the median value (median OS, 12.2 months [95% confidence interval (CI), 8.6–16.6 months] *vs* 5.0 months [95% CI, 3.9–5.7 months]; *P*<0.0001; Figure 1A). Similarly, in the Gem+A arm, patients with a baseline CA 19-9 level equal to or below the median value for that arm lived significantly longer than patients with baseline CA 19-9 above the median value (median OS, 12.5 months [95% CI, 8.6–16.6 months] *vs* 4.9 months [95% CI, 3.6–5.6 months]; *P*<0.0001; Figure 1B). In patients treated with Gem, OS seemed longer among those with a baseline CA 19-9 level equal to or below the median value; however, the difference did not reach significance probably because of the limited sample size (median OS, 11.6 months [95% CI, 3.8–14.7 months] *vs* 5.4 months [95% CI, 3.9–7.7 months]; *P*=0.1109; Figure 1C).

**Figure 2:**
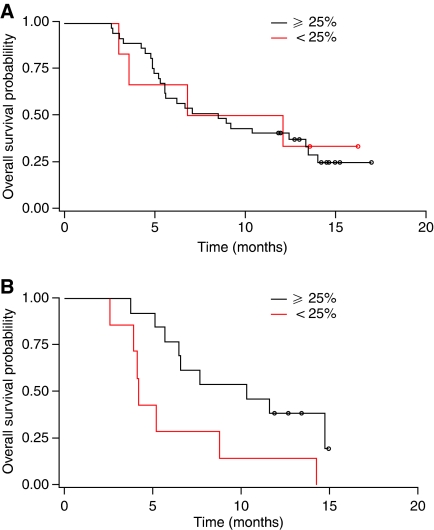
CA 19-9 as a predictor of treatment response: Kaplan–Meier curves for OS according to decline in CA 19-9 value (⩾ and <25% compared with baseline) during treatment with (**A**) Gem+A and (**B**) Gem alone.

**Figure 3:**
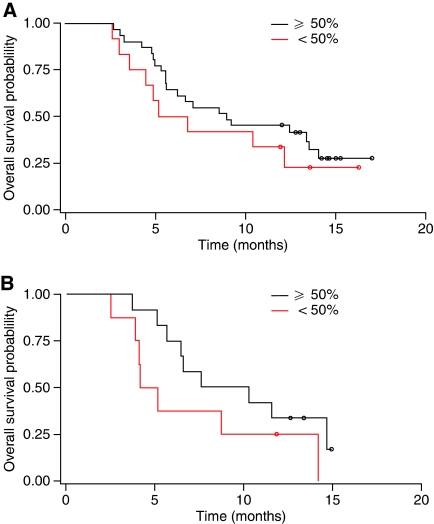
CA 19-9 as a predictor of treatment response: Kaplan–Meier curves for OS according to decline in CA 19-9 value (⩾ and <50% compared with baseline) during treatment with (**A**) Gem+A and (**B**) Gem alone.

For the population as a whole, a non-significant trend to longer OS was observed in patients achieving a reduction of ⩾25% in CA 19-9 levels during treatment than in those patients with CA 19-9 reductions below this threshold (median OS, 8.8 months *vs* 5.2 months; *P*=0.1779), as well as longer PFS (median PFS, 6.7 *vs* 2.5 months; *P*=0.1002). In the Gem+A arm, OS and PFS again showed a non-significant trend, but in the Gem arm, the differences were significant despite the smaller numbers of patients (Table 2; Figures 2 and 3). Five patients receiving Gem+A achieved a confirmed PR per RECIST; four of these patients had a ⩾85% reduction in CA 19-9 levels during treatment and one a 54% reduction. One patient receiving Gem experienced a PR and had a 52% reduction in CA 19-9 levels during treatment.

We also analysed the relationship between dBP, response and serum CA 19-9. dBP seemed to be a strong response predictor in the Gem+A arm, but not the Gem arm. The median percentage drop to the CA 19-9 nadir was 59.9% in the group that had any dBP <90 mmHg, and 61.9% in the group that had any diastolic reading ⩾90 mmHg (Table 3; *P*=0.5201 for difference in medians). Given the small sample sizes, we were not able to detect any statistical interaction between dBP and CA 19-9 for OS. Analysis of kinetic differences, distribution or volatility (by assessing inter-quartile ranges) in the two groups was unable to show any obvious differences (data not shown).

## Discussion

The advances in the treatment of pancreatic cancer have been extremely disappointing. The first series of randomised trials with novel agents targeting specific molecular pathways have not clinically improved patient outcomes. Therefore, a need exists for biomarkers to test in the randomised phase II setting to inform phase III trial design and to enable better understanding of how best to integrate these novel agents into treatment approaches. CA 19-9 has prognostic and predictive value with conventional cytotoxic agents, but little is known about its kinetics with the newer class of cytostatic treatments.

This retrospective analysis is based on prospectively collected data from patients with APC, randomised to receive either Gem+A or Gem (Spano *et al*, 2008). Several molecularly targeted therapies are currently being assessed in patients with pancreatic cancer, but, to our knowledge, this is the first published analysis of its type in patients receiving a potent VEGFR inhibitor that seems to influence OS. We had earlier shown that achieving a dBP ⩾90 mmHg at any time during Gem+A treatment seemed to have great influence on patient outcome: in a *post hoc* exploratory analysis, median OS in the Gem+A group was 13.0 (95% CI, 8.5–16.6) months for the 27 patients with at least one dBP measurement ⩾90 mmHg during treatment in clinic, compared with 5.6 (95% CI, 4.8–7.2) months for the 34 patients without any dBP measurement ⩾90 mmHg during treatment and with ⩾1 blood pressure measurement after cycle 1, day 1 (Spano *et al*, 2008). Patients in the Gem+A group without any dBP measurement ⩾90 mmHg had similar survival to patients in the Gem group.

In our study, CA 19-9 measurements were not centralised, but were performed at each centre independently (ensuring reasonable intra-patient consistency), with no requirement to use a standardised assay. This is a limitation in the context of our retrospective study. CA 19-9 values during treatment were available for only 75 of the 95 patients with baseline CA 19-9 levels. Although this is also a limitation of our analysis, other studies have reported similar or even greater drop-out rates (Maisey *et al*, 2005; Hess *et al*, 2008).

In agreement with earlier published reports (Saad *et al*, 2002; Micke *et al*, 2003; Louvet *et al*, 2005; Maisey *et al*, 2005; Hess *et al*, 2008; Park *et al*, 2008), we found that baseline CA 19-9 levels were prognostic for survival in the whole group; patients with baseline CA 19-9 values at or below the median for the population lived significantly longer than those with baseline values above the median. This was true for the population overall (12.2 months *vs* 5.0 months; *P*<0.0001) and reached significance in the Gem+A arm (*P*<0.0001), but not in the Gem arm, although numerical values were similar. This may be due to longer survival in the Gem+A arm, or possibly because of the lower patient numbers in the Gem arm. Our study also showed that declining CA 19-9 levels during treatment may be associated with favourable patient outcome, but only in the Gem arm. Using thresholds of 25% and 50% reduction in CA 19-9 levels compared with baseline, OS and PFS were both significantly longer in patients receiving Gem with a ⩾25% or ⩾50% decline in CA 19-9 than in those with less decline. In the Gem+A arm, although there was a trend to longer OS and PFS, these were not statistically significant. Little is known about the CA 19-9 volatility on discontinuation of novel drugs. There seems to be rapid rebound phenomenon on discontinuation of vascular-targeted agents such as bevacizumab in other disease settings. One hypothesis is that increased volatility in serum biomarkers when temporarily discontinuing therapy (because of toxicities or lack of compliance) may confound statistical analysis. In this study, we attempted to investigate this possibility, but could not find evidence to support the hypothesis.

Another possibility is that cytostatic agents have a different effect on pancreatic cancer cells. Supporting this hypothesis, patients achieving objective tumour responses, whether treated with Gem+A or Gem, all experienced a decline in CA 19-9 of at least 50% when on treatment. A similar association between tumour response and CA 19-9 decline has been reported elsewhere (Stemmler *et al*, 2003; Ziske *et al*, 2003; Ko *et al*, 2005; Hess *et al*, 2008). Our data concur with those of others that suggest that CA 19-9 decline in the presence of objective radiological responses is potentially a predictive indicator of clinical benefit.

CA 19-9 levels tend to increase with stage of pancreatic cancer (Micke *et al*, 2003; Jiang *et al*, 2004), but vary widely, both inter- and intra-patient (Micke *et al*, 2003; Ziske *et al*, 2003; Hess *et al*, 2008; Park *et al*, 2008). Our study seems to support this, showing an inverse relationship between CA 19-9 and a more advanced phase of disease. Researchers have commonly used the median baseline CA 19-9 level of the study population as a threshold for dividing patients with APC into groups for survival comparisons (Saad *et al*, 2002; Micke *et al*, 2003; Maisey *et al*, 2005; Hess *et al*, 2008; Park *et al*, 2008). The wide variation in baseline CA 19-9 levels makes selection of an absolute threshold for this type of analysis somewhat arbitrary. However, as the data regarding the importance of baseline CA 19-9 as an independent prognostic factor for survival is increasing for a range of treatments (Micke *et al*, 2003; Louvet *et al*, 2005; Maisey *et al*, 2005; Hess *et al*, 2008), an absolute threshold value would be valuable to enable separation of different prognostic subgroups, which in turn will allow prospective determination of prognosis for an individual patient. As discussed by Boeck *et al* (2006), this absolute threshold value could be selected only by conducting a prospective clinical trial that prospectively defines it (e.g. selecting an absolute CA 19-9 threshold of 1000 U ml^–1^ for patients with metastatic disease).

Several studies have shown that an early CA 19-9 response (defined variously as a decrease of >20–50% in CA 19-9 compared with baseline within the first 6–8 weeks of treatment) is associated with significantly longer survival and/or is an independent predictor of survival on multivariate analysis (Halm *et al*, 2000; Stemmler *et al*, 2003; Ziske *et al*, 2003; Maisey *et al*, 2005). In our analysis, CA 19-9 response was not time defined, but an association with survival was nonetheless observed, similar to that reported by Ko *et al* (2005). The median time to CA 19-9 nadir in our study included a wide range (70 days; range, 15–251; and 79.5 days; range, 26–287 on the Gem+A and Gem arms, respectively) and was longer than the 6–8 weeks used in the definition of ‘early response’ as described above. The effect of the difference in definition of CA 19-9 response (time to nadir) on the analyses (association between CA 19-9 response and patient outcome) is unclear. Patients who do not meet RECIST criteria may have a CA 19-9 reduction and contribute to prolonged survival as well as the time to CA 19-9 nadir. Furthermore, the tendency of targeted biologics to prevent tumour progression (i.e. *cytostatic vs cytotoxic* mechanisms), not revealed by RECIST criteria, may confound this phenomenon.

A recent analysis of a large cohort of patients (*n*=247 with baseline CA 19-9 levels; *n*=175 with baseline and at least one follow-up value during treatment) entered in a randomised, controlled clinical trial found no significant difference in survival between patients achieving an early CA 19-9 response (⩾50% decrease in CA 19-9 at day 42) and non-responders (Hess *et al*, 2008). Equally, there was no significant survival difference between responders and non-responders at CA 19-9 nadir; median time to nadir (63 days; range, 7–145) was similar to that in our study, although with a smaller range. The findings from this study, which eliminated guarantee-time bias (i.e. to eliminate the impact of survival time on the classification of responders and non-responders), seem to indicate that there may not be a prognostic link between CA 19-9 response and OS. However, when the same data were analysed using a Cox-regression model, a borderline significant association between CA 19-9 response and survival was noted (Hess *et al*, 2008). A similar analysis conducted in this study confirms this result and highlights that the wide variation in time to achieve CA 19-9 nadirs delimits choosing a specific early time point for analysis.

Despite its frequent use in clinical practice, CA 19-9 kinetics during therapy needs further validation in the context of randomised studies as a potential early response marker. Inclusion of CA 19-9 measurements in phase III trials would be useful to confirm the preliminary data presented in this study. Currently, CA 19-9 may be of use as an additional tool, in conjunction with imaging and clinical assessment of patient condition, to guide treatment decisions in an individual patient. Given the apparent significance of baseline CA 19-9 in patient prognosis, there is a strong case for it to be used as a stratification factor in randomised trials in patients with APC.

In summary, this study has shown a correlation between baseline CA 19-9 levels and survival and confirms its value as a prognostic factor in a randomised phase II setting.

However, although CA 19-9 may be a predictive factor for response to gemcitabine treatment, it is less clear whether it can be incorporated in studies with novel-targeted agents, which are cytostatic as opposed to cytotoxic. This is the first study of its value in a randomised setting with a novel VEGF inhibitor; our data suggest that more work is needed to validate CA 19-9 as a predictive marker with novel agents. We advise caution in using CA 19-9 in phase II settings as a response biomarker to predict efficacy and as an aid in selection of agents for phase III studies.

## Figures and Tables

**Table 1:** Patient baseline characteristics

	**Gem+A (*n*=65)**	**Gem (*n*=30)**
Median age (range), years	65.0 (44–81)	62.5 (36–78)
Gender (male/female), *n*	32/33	14/16
		
*ECOG PS, n* (%)		
⩽1	59 (91)	27 (90)
2	6 (9)	3 (10)
		
*Disease stage, n* (%)		
Locally advanced		10 (33)
	29 (45)	
Metastatic	36 (55)	20 (67)

Abbreviation: ECOG PS=Eastern Cooperative Oncology Group performance status.

**Table 2:** Median overall survival (OS) and median progression-free survival (PFS) according to percentage decrease in CA 19-9 compared with baseline by treatment group

	**Gem+A**			**Gem**		
**Decrease in CA 19-9**	**<Threshold**	**⩾Threshold**	***P*** ^*^	**<Threshold**	**⩾Threshold**	P*
*Median OS (months)*						
25%	9.5	8.6	0.8989	4.2	10.3	0.0290
50%	6.0	9.0	0.3392	4.7	9.0	0.1828
						
*Median PFS (months)*						
25%	12.2	6.3	0.6237	1.8	6.7	<0.0002
50%	5.3	7.6	0.4154	2.2	6.7	0.0370

^*^P-value for difference between median OS (or PFS) for patients with a CA 19-9 response above or below the indicted threshold; thresholds determined by grouping patients in each treatment arm according to the maximum level of CA 19-9 reduction (or response) during treatment (⩾25% decline *vs* <25% decline; ⩾50% decline *vs* <50% decline). A time-dependent Cox proportional hazards model, which statistically adjusts for the confounding effect of time to CA 19-9 nadir on survival, was used.

**Table 3:** Change in diastolic blood pressure versus median percentage drop to CA 19-9 nadir

	**Change in diastolic blood pressure during treatment**	
	**<90 mmHg (*n*=24)**	**⩾90 mmHg (*n*=18)**
Median drop to CA 19-9 nadir (%)	59.9	61.9
Median (95% confidence interval) overall survival (months)	5.6 (4.8–9.2)	12.0 (9.0–14.1)
